# Cyclic Martensitic Transformations Influence on the Diffusion Of Carbon Atoms in Fe-18 wt.%Mn-2 wt.%Si alloy

**DOI:** 10.1186/s11671-017-1978-z

**Published:** 2017-03-16

**Authors:** Vitaliy E. Danilchenko, Alexander V. Filatov, Vladimir F. Mazanko, Viktor E. Iakovlev

**Affiliations:** 0000 0004 0385 8977 grid.418751.e G.V. Kurdyumov Institute for Metal Physics, NAS of Ukraine, Vernadsky Blvd. 36, Kyiv, 03142 Ukraine

**Keywords:** Martensitic transformation, Structure defects, Chaotic stacking faults

## Abstract

A significant carbon diffusion mobility acceleration as a result of cyclic γ↔ε martensitic transformations in iron-manganese alloy is determined by one- and two-dimensional structure defects of ε-martensite with face-centered close-packed lattice. Such defects (dislocations, low angle sub-boundaries of dislocations, chaotic stacking faults) were formed during cyclic γ↔ε martensitic transformations. Peak carbon diffusion coefficient increase was observed under thermocycling when maximum quantity of lattice defects increase was fixed.

## Background

Martensitic transformations implemented by diffusionless shearing mechanism considerably accelerate the diffusion of substitutional and interstitial atoms in reverted initial phase [1–5]. The intensification of diffusion processes was due to the fact that, under the direct and the reverse martensitic transformations a significant number of dislocations, fragment’s sub-boundaries, and deformation twins were formed, so that, in these areas, the atoms are transported more rapidly. At low temperatures (below 0.5 T_melt_), the excess defects in the crystal structure, generated by martensitic transformation, are able to increase the diffusion mobility of atoms in several orders of magnitude.

In the iron-manganese reverted austenite with low energy of packaging defects, cyclic γ-ε-γ transformations caused a chaotic stacking fault accumulation but did not lead to fragmentation of the structure and formation of additional sub-boundaries. A significant difference between the structural condition and the degree of lattice defects in the crystalline phase components that were formed as a result of γ-α-γ and γ-ε-γ transformation, points up the necessity for further studying of the effect of martensitic γ-ε-γ transformations on diffusion behavior in alloys with low-energy packaging defects. The aim of this work is to study the effect of cyclic γ-ε-γ martensitic transformations on diffusion properties of carbon atoms in iron-manganese alloy using radioactive isotopes technique.

## Methods

Investigations were carried out on the Fe-18 wt.%Mn-2 wt.%Si alloy in which, as a result of thermal cycling under γ-ε-γ-transformations, more than 90% of ε-martensitic phase was formed. This allowed to achieve a high degree of hardening phase by γ-ε-γ transformation. Carbon ^14^C isotope was deposited on the polished surface of the sample 10 × 10 × 5 mm with the temperature of 800 °C using a carburizer containing Ba^14^CO_3_ compound. The direct cyclic γ-ε and reverse ε-γ transformations in the ^14^C coated alloy were carried out by periodic cooling in liquid nitrogen and subsequent heating in a salt bath with the temperature of 350 °C. The volume fraction of ε-phase was measured by automated X-ray diffractometer DRON-3 by the ratio of integral intensity of diffraction reflections (111)_γ_ and (002)_ε_ and by the crystallographic factors of repeatability of these planes. After γ-ε-γ transformations, prolonged diffusion annealing of the phase-hardened alloy at temperatures of 100 and 200 °C was performed. After thermal cycling, the alloy at room temperature was in the two-phase γ + ε state.

## Results and discussion

**Fig. 1:**
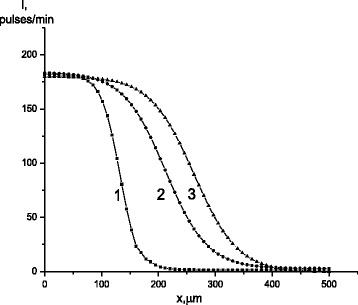
The concentration distribution of radioisotope ^14^C by depth: 1, 2, 3—after 10, 100, and 500 cycles of γ↔ε transformations, respectively

**Fig. 2:**
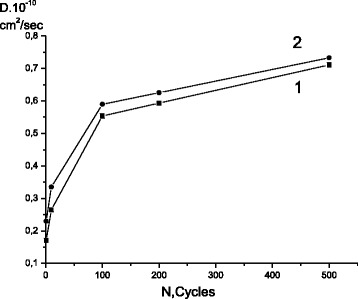
The dependence of the carbon diffusion coefficient on the number of the γ↔ε transformations: 1, 2—diffusion annealing at temperatures 100 and 200 °C, respectively

The depth of penetration of carbon atoms after diffusion annealing increased greatly with the number of previous cycles of transformation, i.e., with increasing the degree of hardening phase (Fig. 1). Calculations showed that, after the first cycle of γ↔ε transformation, the diffusion coefficient of carbon at temperatures of 100 and 200 °C was equal to 1.7 × 10^−11^ and 2.3 × 10^−11^ cm^2^/s, respectively. Increasing the number of γ↔ε cycles (*N*) to 100 led to its growth by a factor of 2.5–3.2. Further increasing the number of cycles to 200 led to an additional, less significant, increase of *D* value and deviation of *D*(*N*) from linear. As a result of 500 γ-ε-γ cycles, the diffusion coefficient at temperatures of 100 and 200 °C increased by 3.2 and 4 times, respectively, (Fig. 2).

For comparison, it should be noted that the growth, Δ*D*, of the self-diffusion coefficient of titanium both in low-temperature α−iron and in high-temperature β−iron, remains approximately linear as we increase the number of cycles of martensitic α↔β transformation [6]. The above-mentioned difference in Δ*D* values for γ↔ε and α↔β transformations in ferromanganese alloys and titanium ones, respectively, is associated with the nature of defects generated by these different transformations.

**Fig. 3:**
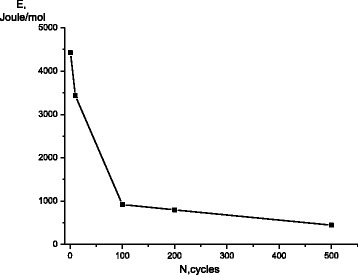
The dependence of the activation energy of carbon diffusion on the number of γ↔ε transformations

The difference between the diffusion coefficient values at 100 and 200 °C was sufficient to determine the diffusion activation energy *E*. The value of *E* decreased steadily with increasing the number γ↔ε transformations (Fig. 3). The main change of *E* (about four times) followed the increase of the number of cycles to 100.

In analyzing the causes of the diffusion mobility growth of carbon in phase-hardened alloy Fe-18 wt.%Mn-2 wt.%Si, we should consider two different mechanisms that make the diffusion possible. The first one develops with the change of the structure of the alloy followed by tension and compression stresses due to the volume effect of martensitic transformation. This mechanism of carbon diffusion is mainly athermal.

Another mechanism of carbon diffusion has been implemented as a result of the diffusion annealing after the cycles of martensitic transformations. This mechanism is defined by the structural features of the alloys state, and, in particular, by additional defects in the crystal structure formed as a result of cyclic γ-ε-γ tranformations. These defects proved to be the paths of accelerated diffusion.

It is known that, as a result of the reverse ε-γ-transformation in ferromanganese alloys, the dislocation density in the reverted austenite raised approximately by one order of the magnitude [7]. For comparison, the reverse α-γ-transformation in iron alloys lead to a significant increase in dislocation density in the initial phase (by three orders of magnitude) [8]. Such γ-α and γ-ε tranformations were accompanied by specific volume increase of 3.4 and 1.75%, respectively.

It was shown experimentally [6] that at temperatures lower than 0.5T_melt_, diffusion of interstitial and substitutional atoms in the bulk of the crystal lattice occurred mainly on dislocations, grain boundaries, sub-boundaries of fragments, and on other defects. This means that the intensification of carbon diffusion after the first cycle of γ-ε-γ transformation is completely determined by the growth of dislocation density in the initial γ-phase. Due to the shear nature of the direct γ-ε and the reverse ε-γ transformations, we can observe the increase in diffusion coefficient of the reverted austenite and ε-martensite formed by subsequent cooling. Further growth in the *D* value as a result of cyclic thermal cycling was insignificant under the low dislocation density growth. In the ferromanganese alloys after γ-ε-γ transformations, the austenite high-angle grain boundaries were not observed, as contrasted with in iron-nickel alloys after γ-α-γ transformations [8, 9].

However, as a result of cyclic γ-ε-γ transformation in the investigated alloy, low-angle dislocation sub-boundaries were observed, that can be characterized by the values of the crystal lattice disorientation. Lattice disorientations in Fe-18 wt.%Mn-2 wt.%Si alloy were measured by azimuthal blurring reflex (101)_ε_ of ε-phase on the diffraction pattern of single-crystal samples. It was found that the maximum angle of disorientation monotonously increased with increasing the number of γ-ε-γ transformations and reached the value of (13-15)° as a result of 80–100 thermal cycles.

Due to high repayment of ε-γ transformations, further thermal cycling did not result in the growth of disorientation angle. It should be mentioned that the disorientation angle did not exceed the value of 15°, which is attributed to high-angle grain boundaries. It is obvious that the disoriented sub-boundaries, as well as other structural defects, can be ways of rapid diffusion.

The X-ray investigations in this study, as well as in previous works [10, 11], have shown that, as a result of cyclic γ-ε-γ transformations in reverted austenite and ε-martensite of ferromanganese alloys with low-energy packaging defects, the chaotic stacking faults were formed along crystallographic plane {001}_ε_. As a result of cyclic γ-ε-γ transformations, the chaotic stacking faults had the ability to accumulate.

These experiments showed for the first time that the phase hardening due to γ-ε-γ transformation, with generation and the subsequent accumulation of chaotic stacking faults, can accelerate significantly the diffusion of interstitial atoms.

## Conclusions

Therefore, a significant acceleration of the diffusion mobility of carbon under the cyclic γ↔ε martensitic transformations in ferromanganese alloy is governed by two different mechanisms—an athermal mechanism determined by martensitic transformation, and the mechanism of thermally activated diffusion by one-dimensional and two-dimensional defects in the crystalline structure of ε-martensite, which are formed in the process of the transformation (dislocations, low-angle dislocation sub-boundaries, chaotic stacking faults). The last mechanism is realized by diffusion annealing of phase-hardened alloy.

Significant acceleration of diffusion mobility of carbon at low temperatures under the cyclic γ↔ε transformations can be used to enhance cementation processes in ferromanganese alloys.
